# Innovative Policy Supports for Integration in Health and Social Care Focused on Culturally and Linguistically Diverse Populations in Australia: A Qualitative Study

**DOI:** 10.1007/s10903-025-01697-8

**Published:** 2025-04-29

**Authors:** Maria Gabriela Uribe Guajardo, Ferdinand C. Mukumbang, Mithilesh Dronavalli, Susan Woolfenden, Lisa Parcsi, Brendon McDougall, James Gillespie, Ilan Katz, Andrew Page, Vicki Giannopoulos, John Eastwood, Michelle Cunich, Carmen Huckel Schneider

**Affiliations:** 1 The Leeder Centre for Health Policy, Economics and Data, Sydney School of Public Health, University of Sydney, Sydney, Australia; 2 Sydney Institute for Women, Children and their Families, Sydney Local Health District, Sydney, Australia; 3 School of Global Health, University of Washington, Seattle, USA; 4 Translation Health Research Institute, Western Sydney University, Sydney, Australia; 5 Community Paediatric Research Group, Central Clinical School, Faculty of Medicine and Health, University of Sydney, Sydney, Australia; 6 Sydney Local Health District, Sydney, Australia; 7 South West Sydney Local Health District, Sydney, Australia; 8 Social Policy Research Centre, University of New South Wales, Sydney, Australia; 9 Drug Health Services, Edith Collins Centre, Sydney Local Health District, Sydney, Australia; 10 Charles Perkins Centre, Central Clinical School, Faculty of Medicine and Health, Boden Initiative, The University of Sydney, Sydney, Australia

**Keywords:** Culturally and Linguistically Diverse, Social Programs, Health Programs, Integration, Policy Supports

## Abstract

The fragmented nature of Australian health and social care systems present a barrier to integrated care. Culturally and linguistically diverse (CALD) communities are recognised as a priority population with unmet health and social needs. This study describes policy supports for programs that promote health and social care integration with a CALD focus, including governance and partnerships; workforce and staffing; funding and payment; and data sharing and use. The research question was ‘what innovative policy supports to integrate health and social programs?’. Qualitative interviews of participants involved in the implementation of health and social care programs in the Sydney using the Innovative Policy Supports For Integrated Health And Social Care Programs Framework, were conducted. Twenty-seven participants from 24 health and social programs based in Sydney participated in the study. Six programs serviced CALD communities only. Ten had majority of CALD clients, with the remaining having a mixture of clients. Ten programs had a formal coordinator role. Most programs did not report new approaches to data sharing. Two out of the 6 CALD targeted programs reported data-sharing via teleconference in the context of emergency. These were 2 health programs addressing COVID-19 disparities and the humanitarian needs of refugees, respectively. Only 2 reported a special funding to assist vulnerable families and common emergency department presenters, respectively. This study demonstrated the lack of integration of services in health and social care. Policy development and implementation should consider bringing stakeholders together (informed by CALD groups) to advance the generation of technology for adopting universal standards and the integration of funding to better support health and social care for CALD communities in multicultural Australia.

## Background

For many years, integrated care policy and programs have focused on primary and hospital care integration [1–3]. The fragmented nature of Australian health and social care systems present a barrier to integrated health and social care; rendering Australia far behind other countries with more mature integrated health and social care systems. Nevertheless, the Australian Federal, and State Government are accelerating integrated collaborations, as well as bottom-up (more common) local partnerships between health and social care to coordinate and deliver more responsive and individual-centred care for individuals from priority populations who are experiencing adversity [4, 5].

Australia is an ethnically diverse nation with three million permanent migrants resettled since 2000 [6]. Of these, almost 90% are living in State capital cities, with Sydney Metropolitan being the most common place of settlement [6]. The term culturally and linguistically diverse (CALD) is commonly used in Australia to describe people born in non-English speaking countries where English is not the primary language spoken at home (including second-generation family members) [7]. From a health policy perspective, CALD communities are recognised as priority populations with marked unmet health and social needs [8] along with systems barriers [6]. 

Specifically, refugees and humanitarian entrants are more likely to self-report certain long-term health conditions, including diabetes (80% higher), kidney disease (80%), stroke (40%) and dementia (30%) than the rest of the Australian population [9].

In addition, CALD groups, especially those with a humanitarian background, have greater levels of adversity compared to other groups [7]. Under The National Settlement Framework, the three tiers of Australian Government, Commonwealth, State and Territory and Local Government, work in partnership to effectively plan and deliver services that support migrants and new arrivals in Australia [10]. The supports include language services, employment, education, housing, health and wellbeing, transport, civic participation, family and social support, and justice [10].

In addition, and beyond the settlement years (post 5 years), integrated locally health and social care support (in ad-hoc programs) is still needed to support CALD community needs.

This qualitative study sought to find and describe policy supports for programs that promote health and social care integration as they are emerging at local levels in the Australian system; specifically analysing four broad categories: governance and partnerships; workforce and staffing; funding and payment; and data sharing and use. The study focussed on health and social care programs, supporting individuals experiencing adversity with a particular focus on CALD populations, based in the Sydney Metropolitan region. The research questions are: to what extent do collaborative health and social programs display (i). new governance structures or collaborative partnerships?, (ii) joint staffing models for health and social care delivery, or new roles and responsibilities for staff? (iii) joint financing of health and social care by changing the way that funding for the program is provided?, and (iv). processes for health and social care providers to jointly collect or share data?

## Theorical Framework

A framework for understanding the structures underpinning integrated health and social care generation and implementation in local systems, has been developed by Wodchis and collaborators [11] based on combining several integrated care policy research frameworks [12–15]. More broadly, we explored the degree to which local health and social programs (health care, social care, NGOs, community organisations, volunteers organisations) innovated, facilitated and advanced integrated health and social care knowledge, practice, and models of care at local levels [11].

The description of each category of the framework are presented in Table 1.

**Table 1:** Framework based on Wodchis et al. 2020

Category^1^ Number and item	Description
Category 1: Integrated governance, oversight and collaborations	Characterised by a unique form of governance or new collaborative partnerships between organisations in the health and social care domains. Programs can also be reported to have had significant changes in the governance structure of local health care systems, the extent of local collaborations required to establish and implement the programs or both.
Category 2: Integrated health and social care workforce and staffing requirements	Novel approaches undertaken to filling staff requirements and work roles are implemented. Broadening the roles of health and social care providers, creating new work roles, or developing new ways of working for existing health and social care providers. Programs with defined supportive workforce or staffing policies with new local efforts to have health and social care providers work jointly, with or without adding any new staffing roles or the creation of multidisciplinary team-based approaches.
Category 3: Integrated financing processes and payment methods	Recognised changes made to financing and payment policy as necessary supports for the integrated model. This may involve the creation of new budgets to ensure the entire cost of the health and social care services for the target populations is achieved. Total or combined budgets are established, new envelopes of funding for more centralised programs, and agreements to share the risk associated with delivering the integrated care among health and social care organisations including insurance companies and private health funds can also be mapped.
Category 4: Integrated data sharing and best usage of those data	Novel approaches to generating required data or information technology solutions. This may involve sharing patient information with one group (provider) to have access to the clinical records of another group (provider). Other forms may include staff sharing information about patients across the providers involved in delivering the integrated model. Secondary uses of data include integrated programs creating standardised reports about the progress of the integrated care program (such as the number of patients enrolled, usage, statistics on key clinical or social indicators) which are consistent with current approaches to monitoring programs or programs engage third-party external (such as university employees) groups to develop, undertake and maintain data and describe key outcomes of the integrated care program.

^1^ All categories sourced based on Wodchis et al. 2020 [12]

## Methods

### Participants

The study’s research team is part of a research centre (Centre for Research Excellence in Integrated Health and Social Care) partnering with local health districts and other key stakeholders. This allowed first in contact with health and social care programs throughout research and services’ network. A snowballing method was the used to branch further out into other health and social care programs through common and known participants.

### Recruitment

The study was advertised in the health and social care service networks, by sending an expression of interest email (EOI) to several health and social agencies that provide health and social services in the Sydney Metropolitan area. Program representatives were volunteers who contacted the principal investigator (MGU) for enrolment and other information. Programs representatives were eligible to participate if they were assisting priority populations, including CALD) communities, or programs that have a role (funding, liaison, advocacy) assisting health and social care programs (e.g. Ministry of Health).

### Data Collection and Measures

Participants were involved in the implementation of health and social care programs in the Sydney Metropolitan area either as administrators, service providers or volunteers and/or community leaders. Health and social care programs were defined broadly and not identified a priori.

Demographic information was collected during the interviews. These semi-structured interviews explored innovative policy supports were conducted online (via video conference) by the first author (MGUG) and lasted approximately 45 min each. They were conducted from June 2023 to April 2024.

### Analysis

Demographic descriptive analysis was performed using IBM SPSS Version 29. The thematic analysis and coding involved a team of authors (MGUG, CHS), who were experts in qualitative technique (several meetings were held to endorse themes and structure of this work).

Using the interview, it was determined the CALD intake of each program, categorised them as follows:

1. CALD-specific: programs serving only CALD clients (100%).
2. High intake: programs serving 50% or above CALD clients (open to non-CALD Australians).
3. Moderated uptake: programs serving below 50% CALD clients (open to non-CALD Australians).

### Ethics Approval

The study was approved by the Ethics Review Committee ( RPAH Zone) of the Sydney LHD (protocol number X22-0406) and the South Western Sydney LHD Human Research Ethics Committee (2023/STE00775). All participants provided written informed consent.

## Results

Health programs, delivering health treatment and services for maintaining health, including Local Health District (LHD) level services (e.g. outpatient, health promotion, population health, drug health service staff), Primary Health Network led or commissioned programs (PHN), Ministry of Health (MoH) were analysed against the four categories devised by Wodchis et al. [11].

Social programs that focused on the social and economic wellbeing of the priority populations such as income support and social and economic employment-related programs and services, legal aid, domestic violence services, adult and youth community support programs in Sydney Metropolitan were also assessed.

Twenty-seven participants from 24 programs (14 health programs; 10 social programs) based in metropolitan Sydney were part of the study. Interviews were conducted with clinical directors, health and social service managers, researchers implementing programs, staff specialists (e.g. paediatricians, public health physicians), and community leaders serving CALD clients. 71% were female. The mean age was 50.18 years (SD = 12.36). 42% of participants were from an ethnic diverse background (e.g. Arabic, Chinese, Vietnamese, Macedonian, Spanish). The most common background was Australian.

Of the 14 health care programs, 2 (MoH; PHN) provided system support to health systems without service provision. Of the 10 social care programs, only one provided system support without service provision (Legal and Health Enhancement).

There were six (both health and social) programs developed to serve CALD communities, and 10 programs with high intake of CALD clients with the remaining having a mixture of clients. Table 2 presents the details of the programs part of this study.

**Table 2:** Programs’ descriptives

Label	Service type	Population
Family CALD Program # 1	Health promotion	CALD specific
Family CALD Program # 2	Health promotion	CALD specific
Family Refugee Program # 3	Health screening and referral pathways and coordination	Refugees and asylum seekers specific
Women’s Program #1	Healthcare service delivery	Women*
Women’s Program # 2	Counselling service delivery	Vulnerable women**
Children Program # 1	Healthcare service delivery and coordination	Rural children with chronic conditions accessing metropolitan services**
Children Program # 2	Healthcare service delivery and coordination	Children and young people with cerebral palsy**
Children Program # 3	Health and social care delivery and coordination	Pregnant women who are Department of Community and Justice clients, including children and babies*
Family Program # 1	Healthcare service delivery and coordination	Women, children and their families experiencing adversity*
Children Program # 4	Health assessment and referral service	Foster care children*
Adult Program # 1	Healthcare service delivery and coordination	Patients with chronic and complex illness*
Children program # 5	Health and social care coordination	Children and their families experiencing adversity*
Primary Health Program # 1	Comprehensive support to clinical practices	No service provision
Ministerial Organisation # 1	Funding and governance support	No service provision
Youth Program # 1	Social care service delivery	Youth experiencing adversity*
Women’s Program # 1	Social care service delivery	Women experiencing domestic violence*
Family Program # 1	Social care service delivery, linking services and coordination	Families and school-age children*
Adult Program # 1	Social care service delivery	Individuals experiencing adversity*
Adult CALD Program # 1	Social care service delivery (with health components)	CALD specific
Adult CALD Program # 2	Social care service delivery (with health components)	CALD specific
Adult Program # 2	Social care service delivery	Individuals experiencing adversity**
Legal and Health Enhancement	Policy advocacy, and systems change (legal and health partnerships) for vulnerable groups	No service provision
Adult CALD Program # 3	Social and health care service delivery	CALD specific
Adult Program # 3	Legal and social care and primary care linking	Individuals experiencing adversity*

CALD: culturally and linguistically diverse

*High intake of culturally and linguistically diverse clients

** Moderate intake of culturally and linguistically diverse clients

### Analysis of Specific Supports

This study systematically explored four different policy supports as presented below. It explored the creation of new governance structures or new collaborative partnerships between health and social programs; creation of new staffing models for health and social programs’ delivery, redefining, or creation of new roles and responsibilities for staff; new ways for health and social programs providers to collect or share data in a timely fashion; and new ways to finance health and social care by changing the way that funding for the program is provided or the way that the providers of care are paid.

### Partnerships and Shared Governance Policy Supports

Of a total of 24 program, 15 (11 health; 4 social) reported that they have been supported by the establishment of new governance structures between existing health and social care authorities; most commonly in the form of steering and board committees. For instance, interview #022 participant described ‘*our service sits under the violence*,* abuse and neglect directorate and we are part of steering committee that assesses the different services and how services are responding to the needs of its clients with involvement from the Ministry of Health and Out of Home Care Programs Statewide’.*

Four (health) programs reported having steering committees with informal partnership with the social care sector. Pilot health research programs (n = 3) were reported to have established partnerships. They were composed and purposively designed for active and connected engagements aligned with research and implementation milestones.

Interview # 012 participant highlighted the existence of *‘an overarching steering committee for our initiative comprised of researchers*,* academics*,* primarily*,* and key individuals involved in implementation*,* like myself. We have also second local internal steering committee*,* comprised of typically managers*,* and the clinical teams that operate within the centre as they oversee and have discussions about the ongoing implementation of the initiative’.*

Only two (health) programs reported formalised structure and accountability where all the relevant stakeholders have clear responsibilities for the client’s care and their outcomes. These were the Out of Home Care Program and the Perinatal Conferencing Program which reported to have joint governance with stakeholders from the LHD and Department of Community and Justice (DCJ). It is important to note that referrals to both programs are made by DCJ within 14 days of the Children’s Court order allocating interim parental responsibility to the Minister.

Three programs (2 health;1 social) were entering partnerships or collaborations to navigate social and health crises.

Further, a health provider assisting refugees, reported the creation of a nationwide support network to prepare for Ukrainian humanitarian entrants to Australia, with settlement services, and the Department of Education. For instance, interview #020 participant described emerging partnerships: ‘*Different stakeholders nationwide in the refugee space came together*,* medical*,* social services*,* settlement services*,* Department of Education to discuss new arrival trends (Palestinians and Ukranian) to coordinate/provide the case management*,* and casework support to new refugee influx’.*

Another health program (State level), created a Local Emergency Management Committee to manage COVID-19 pandemic, by partnering with the DCJ, Department of Education Training (Primary, Secondary and Tertiary), Health State Emergency Operations Centre. This partnership functioned as a ‘Hot Line’ with the vision to provide high-level information and decision-making to all sectors. For example, Interview #027 participant described: *‘When this pandemic looked like it was going to be a big*,* bad and nasty one*,* I moved from my usual role*,* which is doing all sorts of other things for the Chief Health Officer at the Ministry to take on a senior role liaising with Department of Community Justice*,* Department of Education and Training*,* and also with the whole of health state emergency operations centre. This meant we formed a partnership as ‘hotline’ with Secretary of the Department of Communities*,* and Justice at the most senior levels*,* get information to those very important public servants*,* including for the Head of Primary School (including childcare) Secondary and University sectors’*.

### CALD Specific Programs

Five out of the 6 CALD-specific health and social programs evidenced partnerships across sectors.

### Integrated Health and Social Care Staff Policy Supports

A total of 10 health and social programs have supportive workforce to have health and social care providers work together in a coordinated manner. These reported having a formal coordinator role, who is not jointly (health and social care) employed, or funded.

The term utilised to define ‘coordination’ was heterogeneous. The labels to define coordination included ‘service navigator’, ‘hub navigator’, ‘coordinator’, ‘social prescriber’, ‘community linker’, and ‘hub leader’.

For example:

Two programs reported (1 health; 1 social) adopting a ‘wrap round model’ that provides individualised care using an array of formal services and natural supports, as an alternative when addressing clients with complex needs, especially those experiencing domestic violence and vulnerable families. Interview #018 participant described *‘The team started to do intensive wraparound on almost every client*,* performing this case management wraparound model*,* which is part of what their success was actually because the care navigation approach would not have really worked initially’.*

In addition, one of the programs reported using a ‘caregiver’ to coordinate and support care management, as a key inclusion requirement for individuals with high number of emergency presentations and hospital admissions.

One health program reported the use of ‘high-level (health) liaison role, that coordinated emergency public health response bringing different departments (social and health stakeholders) together to develop a system-salient State plan to mitigate COVID-19 negative outcomes.

### CALD Specific Programs

Three out of the 6 culturally specific health and social programs reported employing coordinators to provide system navigation to CALD clients.

### Data Sharing Policy Supports

Most programs did not report being supported by new approaches to data sharing or joint information technology. Of the 24 programs, none had an established secure electronic system to share client data in real-time with other (external) health or social services and their partners, respectively. Three health pilot research programs created their secure electronic database to capture health outcomes for clients entering the program to external evaluation (with steering committee oversight for research practice and data appraisal).

Of the programs that served clients directly (21 programs), secondary data was used to create standard reporting on key program outcomes (such as number of clients enrolled) mainly for annual funding reporting, which mirrored existing approaches to data monitoring. For instance, interview #010 participant reports: *‘We have to report to the different departments involved regarding the statistics*,* how many clients we have served*,* how many clients have Arabic background*,* details of clients needing that particular service for that particular help as this is required from funders’.*

Where shared viewing was not enabled, there was a reliance on specific people to share information about clients across providers. The most common way to share client information among providers from different sectors was the use of conferencing or online telephone meetings for interagency and cross-sectorial work.

Importantly, one of the main barriers to intersectional data (e.g. same local health district) sharing is the lack of interoperability. For example, Interview #013 participant reported *‘Sometimes when we have to do referrals to oral health*,* they’ve got a system called Titanium but this system is not compatible with our one*,* and they don’t share records with us’.*

### CALD Specific Programs

Two out of the 6 programs reported data-sharing via teleconference in the context of emergency. These were 2 health programs addressing COVID-19 ethnic diverse disparities and the humanitarian needs of refugees, respectively. For example, Interview # 027 highlighted their role as: *‘I took a senior role liaising with Department of Community Justice*,* Department of Education Training*,* and also with the whole of health State emergency operations center*,* including supporting CALD communities in South West Sydney. They had me as a hotline for the most senior levels*,* getting information to those very important public servants*,* as well as education’.*

### Funding System Policy Supports

No aggregated or bundled budgets (where all related health and social care services are pooled in a single fund) were reported. Of the 24 programs studied, only 2 health initiatives reported separate funding pathways as the overall funding arrangements, and thus formed a continuum of approaches to funding and payment in support of innovation and sustainability in the integrated health and social care space. These were centralised policy supports with new budgets created to cover the full cost of all health interventions (with a social care focus) for the target populations, namely, women, children and their families and common emergency department presenters at an LHD level. About one of these initiatives, interview # 020 participant reported that ‘*The original grant was a partnership grant*,* followed by a New South Wales Health grant*,* and Federal funding via primary health network*,* supplemented by from Department of Community paediatrics (Sydney Local Health District). At present*,* this initiative is fully funded directly by New South Wales Health recurrently’.*

Most programs reported the use of a combination of health and social care funding to support staff allocated specific projects (separate pots of funding from health or social care sector). Only two programs reported funding from 2 sources (NSW Health and DCJ). This pooled fund was then used to provide a wide range of health and social care services for pregnant women, mothers and their children under the Out of Home Care Health Pathways Program and the Perinatal Family Conferencing Program.

Interview #022 participant reported: *‘Out of Home Care is health funded by NSW Health and Perinatal Family Conferencing is jointly funded by NSW Health and DCJ’*.

Of note is the fact that there is additional culturally-specific funding for CALD populations beyond welfare, from Federal and State agencies such as Department of Home Affairs (e.g. Settlement Program), and Multicultural NSW (e.g. language programs, community hubs), NSW Health (diversity health promotion services), and Department of Health and Age Care (CALD Living with COVID-19).

The programs studied did not include at-risk funds, payable on the achievement of performance targets nor did they have allowed/added supplementary payments for new services associated with the programs. In addition, none of the programs were supported by highly flexible local funding and payment policies (e.g. discretionary funds to extra cover costs outside the usual care). A very common form of indirect financial support was in kind, through the allocation of staff to clinical research practices (research nurse, health staff being part of steering committees) supported by program-specific funding.

### CALD Specific Programs

Three health programs were funded by health, two social programs were funded by a combination of health and social funders, and one program was run and led by volunteers.

## Discussion

The study sought to assess policy supports for health and social integration in services supporting individuals experiencing adversity with a particular focus on CALD populations. The study analysed governance and partnerships, workforce and staffing, funding and payment, and data sharing implemented in health and social care programs located in metropolitan Sydney [11].

One of the main learnings from this study indicates that cultural-specific programs are more likely to implement flexible innovations (mainly informally) to support migrants and refugees which could be directly linked to emergency context they experience. Forming partnerships in the local context was the most common form of innovation. While still fragmented systems, more than half of the health- and social programs, reported having formal and informal partnerships as core components. However, the number of local shared and formalised integrated health and social care partnerships remains low compared to mature systems in other countries [12].

Australia, as a nation, is far more experienced in creating formal partnership pathways linking primary and hospital health care– than establishing partnerships between health and social care [2, 3, 13, 14].

We found a number of local-level partnerships; that tended to be formed ad hoc, bringing inherent challenges particularly related to sustainability and longer-term effectiveness. It is understood that building meaningful and strong partnerships requires a high level of partnership synergy, involving the availability of resources (funding, equipment, information, skills), relationships amongst partners (e.g. trust, power differentials), partner (e.g. level of involvement) and partnership characteristics, (e.g. governance, efficiency) and external environment (e.g. community characteristics) [15].

Our study demonstrated the existence of formal ‘coordination roles’ (variety of labels) to help high complex clients navigate complex systems. Further, new narratives are emerging in our local context. Innovative programs are now labelling the coordinator role as ‘social prescriber’, a term derived from more mature integrated health and social care systems [16].

In terms of data-sharing practices, our results evidenced the lack of investment in electronic medical records and real-time data sharing as a way of addressing inefficiencies in health and social care systems, particularly in systems of care for high-needs and high-cost populations. The programs surveyed reported a lack of infrastructure, strategy, skills, and resources to collect and share data effectively intersectoral, and across systems which is a global challenge [17]. Effectively, and like other systems, it was reported that often data sharing is supported by other means such as real-time interagency conferencing or online or telephone meetings all crucial when managing clients with complex presentations [17, 18]. New funding policies and innovative funding models were also limited. However, there were some exceptions, like the introduction of pooled funding with integrated governance and responsibility shared equally for health and social care systems. In addition. given the pressing needs of permanent migrants (including humanitarian entrants to Australia) culturally-specific funding beyond welfare, from Federal and State agencies was available, which is often underreported or overlooked.

Limitations of this study should be noted. A clear limitation of this study is the lack of engagement of CALD representatives in the design of the study, scope (relevance and research question), methodologies and data appraisal. A future significant line of work would involve establishing a CALD representative governance that can support culturally salient research projects at a local level. While we aimed to conduct a thorough mapping study that encompasses all the services in Sydney Metropolitan, we acknowledge not all services working with CALD populations were interested in participating given the voluntary nature of the study.

Strengths of this study include capturing the voices of key stakeholders, namely CALD representatives (leaders and volunteers as research participants), health and social care decision makers and staff regarding the state and depth of social and health care integration, local knowledge and evidence that is lacking from the literature.

## Conclusion

This study demonstrated the lack of meaningful integration of services in health and social care. Policy development and implementation should carefully consider ways of bringing stakeholders together (informed by CALD groups) to advance the generation of technology for adopting universal standards and the integration of funding to better support health and social care for CALD communities in multicultural Australia.

## Data Availability

No datasets were generated or analysed during the current study.
